# Report on Therapeutics

**Published:** 1878-08

**Authors:** J. T. M’Millan

**Affiliations:** Paris


### THE DENTAL REGISTER.

VOL. XXXII.] **AUGUST, 1878.** No. 8.

### REPORT ON THERAPEUTICS.

*Read before the Kentucky State Dental Association.*

BY J. T. M'MILLAN, D. D. S., PARIS.

As a member of the committee on therapeutics, I had not fully determined to read a paper, but prompted by the feeling that every member of this Association should endeavor to contribute something in the way of personal experience, and with the knowledge that it is the expectation of the Association to have every one that is appointed on a committee to produce and read before its body an article citing personal experience in the use and application of remedies that may have come under notice. I will in a very brief way, attempt to give the members present, the benefit of a limited experience, although I don't know that I will be able to give remedies and the application of them so much as we would call the attention of the Association to what in the greatest number of instances produces neuralgia, for not until very recently has it become a question for dental investigation let alone to prescribe in these cases. I might name over the great mass of remedies that we find laid down in general therapeutics and materia medica, and still be calling to notice things that have not been personally applied, for by actual experience alone can we be certain that our remedies or therapeutic agents will accomplish what has been marked out for them in general therapeutics. If these remarks only tend to elicit discussion on the part of the members we will feel that much of our aim has been accomplished.

Now as to neuralgia, the word in the most simple English term is indicative of pain, and has particular reference to the nerves and nerve connections.

Webster in his definition of the word gives it of Greek origin, and signifies, nerve pains, continued or intermittent, and something that follows out the entire branches of nerve connection, and thus we find this troublesome disease when it comes more directly under our notice. Cases of neuralgia that are oftenest brought before us, are attributable in the majority, to some local cause or disturbance having been produced by negligence on the part of the patient to observe the necessary precaution of once or twice a year paying a visit to the dentist. It has been the case in our limited observation that we find the greater number of those who suffer from this malady to be such as prefer to contribute dollars to the pockets of our M. D.'s rather than spend the same number of dimes with the D. D. S.'s. We will of course attempt only to speak of such cases of neuralgia as effect that part of the body that we have directly under our charge, and to cite such cases as we may think of interest to those who will give us their attention.

To have any particular treatment of neuralgia we must first have some cause that has produced this trouble in some particular part of the organism. The most common of all the different parts effected by this complaint, is the head, face, shoulders and neck, and those that we have been called upon oftenest to lend our aid to, have been as we stated above, parties who either through a fear of a little suffering at the hands of the dentist or through a greater fear lest they would spend a few dimes of their hoarded wealth, have so neglected their teeth until we find before us patients. and especially ladies, whose ages run from twenty-five to forty-five years, that have been suffering from year to year, and at times suffering the most excruciating pains and have called upon Drs. A., B., C. and D., M. D.'s and taken Quinine, Aconite, Belladonna, Valerian and Morphia, and divers other prescriptions that have so emaciated and broken down their digestive functions and disordered their stomachs that they have had to resort to the use of ferri tonics to restore them to health and vigor again, and now at last they find they will be compelled to visit the dentist. The iron and palliative remedies have done their work, and these very remedies worked destruction to the teeth, and their surroundings have vitiated the secretions, retarded the healthy circulation in the soft parts, etc., and we have a patient broken down in constitution, emaciated and pale, a mouth full of diseased roots and teeth, sore and foul, and from this condition arises disease in all the nerve connections and even those in the associated parts. Continued or intermittent pains in the head, face, shoulders, neck and along the whole vertebra, or as you may have it, chronic facial neuralgia, there is nothing that you can administer that will give more than momentary relief. The whole of the facial nerves have been impregnated with this disorder, and the disease has worked its way to the most remote ramifications of the nerve fibril, and its effect is being vibrated throughout the whole nerve connection, the same as the play of electricity over a telegraph line and it is not always felt most at the part effected, or where the cause is located, but the pain is generally greatest at points of interference or at points of bifurcations of nerve connections. This interference is caused by swollen conditions of the parts connected producing engorgement of the circulation, congestion of the blood vessels, etc.

We have had several just such cases. Our treatment is first to remove all diseased teeth from the mouth, breaking up the primary cause of the trouble, but this does not always end the suffering in all cases, for the sting of the disease is still there. The cause removed it is but a short time before nature begins to assist the remedies to work good to the patient. I have known such patients to suffer for months after the extraction of teeth, because every slight cold or on every exposure to the weather, would stir up anew the disease, the effect of which was still lurking in the nerve tissues.

If we find that what the M. D.'s call the dentist's prescription, (the forceps) does not relieve this neuralgia, we then resort to systemic treatment. I say we and that word is well applied here, for I have invariably called in consultation the family physician of the patient under treatment when I found that systemic treatment had to be resorted to, and I am indebted to an old physician of our place for this formula, and we will not pretend to describe its therapeutic principles, but give it as it is used by him, and will leave it with the association to extract the medicinal effect of the two ingredients, one-fourth of a milligramme or 1-260 grain of tinct. Aconite root, taken in a large wine glass of port wine twice a day. He says he has found quicker relief by using this small quantity of Aconite in connection with the port wine than if he had used tinct. Aconite in a greater quantity. We also use for a local application where the pains are confined around the branches of the fifth pair nerves:

| | | |
|---|---|---|
| ℞ | Chloroform, | ʒiij |
| | Tr. Aconite, | ʒj |
| | Ess. Peppermint, | ʒiv |

Again our attention is drawn to another character of trouble that is in nearly every case producing facial neuralgia, (I say facial in this case for it seems to effect the facial nerves and the pains are felt more in them than they are in the posterior dental nerves, although the dental nerves are more directly effected) and in my immediate locality I find a great many sufferers from it. It is something that has never come directly under the care of the dental surgeon although frequently effecting the soft parts of the mouth and not unfrequently producing severe pains in the teeth; it is what is known as "nasal catarrh."

I had a case a few weeks ago, a lady aet. twenty-six, who had suffered for about two years with almost constant pain in the left side of the face and between her eyes; had on a number of occasions had her teeth examined by her physician, but had found nó cause to produce so much trouble; had resorted to the narcotić effect of morphine to get relief, she had suffered so much, that she had taken as much as one-quarter ounce Morphine a week. Her physician brought her to me for the purpose of having the entire set of teeth extracted, hoping by this to get some relief. She had been in good health, had suffered from no disease; had experienced no trouble except from this side of her face; in masticating the teeth had never given any pain. Upon examination, I found but one carious tooth in her mouth. Never had but few and they had been filled and were in good normal condition the decayed one was the left superior lateral with nothing more than a superficial cavity; in sounding the teeth for soreness found but one, and that first superior molar in the diseased side which gave any signs of trouble and that only produced the slightest pain under severe concussion; the fauces and throat presented a red excited appearance, as if she had been suffering from some disease of the throat. I refused to extract any of the teeth, and requested her to return again in a few days that I might make a more satisfactory examination. She did so; upon a close examination, I found as stated above no decayed teeth with the one exception, but pressure brought to bear on the face just above the canine fossa produced pain and likewise pressure by placing the finger and thumb over the nasal bone produced violent pain, and a somewhat smothered feeling, although she had never discovered this before. By the aid of the speculum I succeeded in dilating the anterior nares sufficient to bring to light the seat of the disease. The whole of the mucous secretions appeared in a state of inflammation and discharging a watery fetid pus or serum; the inflammation seemed to extend into the left maxillary sinus. After watching the case for several days, I determined to extract the molar referred to above so as to get in to the antrum. After extracting found the anterior buccal root very much diseased but posterior buccal and palatal roots presented a normal appearance. Opened up the cavity which did not penetrate the antrum; when the antrum was penetrated there was a discharge of bloody pus, and the whole of the secretions seemed in a state of excitement although there was no discharge in the antrum except at the depression over the root specified: after treating in the usual way with tepid water and a dilute solution of tinct. Calendula, the trouble was soon relieved in the antrum. I can not attribute the disease in the antrum to any other cause than that the patient had been in the habit of when asleep laying on the diseased side which had run the discharge thrown off from the inflamed catarrhal affections through the maxillary sinus into the antrum which could not be discharged, and there created the inflammation which caused the loss of the molar tooth. I prescribed in this case the local application of Chloroform, Aconite, and ess. Peppermint when suffering sever pain, and gave the nasal treatment up to the physician. She suffered intermittently with neuralgia until the catarrhal affection was cured. I have every reason to believe that reflex influence from catarrh, diseased roots of the teeth, diseased antrum, the eruption of wisdom teeth, ulcerated gums, etc., are the causes of, and produce more neuralgia than anything we will ever be able to cite. I believe that a subject suffering from neuralgia if properly studied can be relieved by taking the ordinary remedies used in treating nervous diseases, just so soon as the point of irritation is discovered and removed. Wherever we have a cause that produces trouble and disease to the parts, we are sure afterwards to have the effect although it may not be felt right at the point of irritation.

